# Acute changes in ankle dorsiflexor strength and fNIRS-Derived cortical activation following a single session of neuromuscular electrical stimulation in healthy older adults

**DOI:** 10.3389/fragi.2026.1726632

**Published:** 2026-06-15

**Authors:** Yingqi Li, Luyi Wang, Congxiao Wang, Hujun Wang, Shaoting Zhang, Anda Xiu, Shengxuan Duan, Yingpeng Wang, Shuyan Qie

**Affiliations:** 1 Department of Rehabilitation, Beijing Rehabilitation Hospital, Capital Medical University, Beijing, China; 2 School of Beijing Rehabilitation, Capital Medical University, Beijing, China

**Keywords:** ankle dorsiflexion, cortical activation, functional near-infrared spectroscopy, muscle strength, neuromuscular electrical stimulation, older adults

## Abstract

**Background:**

Neuromuscular electrical stimulation (NMES) is widely used to improve neuromuscular function, but most studies in healthy older adults have focused on longer-term outcomes such as muscle strength, mass, or architecture. Whether a single NMES session can induce measurable immediate changes in ankle-related neuromuscular performance and task-evoked cortical activity in healthy older adults remains unclear.

**Methods:**

Twenty-seven healthy older adults participated in this within-participant pre-post design study. A single 20-min NMES session was applied to the right tibialis anterior (40 Hz, maximum tolerable intensity, 2 s on/10 s off), with intensity individualized to maximal tolerable contraction (mean ± SD: 28.5 ± 6.2 mA; range: 18–42 mA). Before and after NMES, maximal ankle dorsiflexion performance was assessed on a dynamometer, and passive muscle biomechanical properties were quantified with MyotonPro. Whole-brain fNIRS was recorded during the dorsiflexion task. HbO signals were analyzed using a GLM with channel- and ROI-level inference, effect sizes, and false discovery rate (FDR) correction.

**Results:**

NMES increased ankle dorsiflexion strength immediately after stimulation, whereas passive muscle mechanical properties showed no measurable short-term change. Task-related cortical activation was observed in both the pre-intervention and post-intervention sessions. Although exploratory analyses suggested some localized post-pre differences, no cortical effects survived correction for multiple comparisons. Exploratory brain-behavior correlations were observed, but none remained significant after correction.

**Conclusion:**

A single session of NMES improved ankle dorsiflexion strength immediately in healthy older adults, while passive muscle mechanical properties did not change over the same period. Cortical activation was observed in both sessions, but no pre-post differences survived correction for multiple comparisons. The strength findings support an acute neuromuscular effect of NMES, whereas the cortical findings remain inconclusive.

## 1 Introduction

With the accelerating global population aging (Doody et al., 2023), healthy aging has become a widely concerned social issue. Age-related decline in muscle function is a key physiological change during aging, primarily manifesting as reduced muscle strength and impaired endurance (Yoshida S. et al., 2023). This not only impairs older adults’ abilities in activities of daily living such as walking, standing, and climbing stairs, but also increases the risk of falls, fractures, and other health issues, severely compromising their quality of life (Cruz-Jentoft et al., 2019). Therefore, exploring effective interventions to maintain and improve muscle function in older adults is of significant practical importance.

Neuromuscular electrical stimulation (NMES) refers to the application of electrical currents to peripheral nerves or muscles to elicit muscle contractions and augment sensorimotor input. As a rehabilitation and training modality, NMES is commonly used to enhance neuromuscular function (Borzuola et al., 2023a). In the 2017 Clinical Practice Guidelines for Knee Stability and Movement Coordination Impairments, NMES was rated as Grade A evidence and strongly recommended for improving quadriceps strength during anterior cruciate ligament (ACL) rehabilitation (Logerstedt et al., 2017). In stroke rehabilitation, a meta-analysis reported that mirror therapy combined with NMES (MT + NMES) produced greater improvements in lower-limb motor outcomes than control interventions (Oh et al., 2023). More broadly, NMES has been applied to support strength maintenance or functional recovery across rehabilitation contexts (Bondi et al., 2021; Kristensen et al., 2021; Moezy et al., 2024; Othman et al., 2024). Proposed mechanisms include non-physiological motor unit recruitment and synchronization, changes in neuromuscular transmission, and strong afferent sensory volleys that may transiently modulate sensorimotor processing (Maffiuletti, 2010; Carson and Buick, 2021).

Maximal strength is determined not only by muscle morphology but also by the ability to generate adequate neural drive. Therefore, the central effects of NMES are relevant to strength-related outcomes. Age-related weakness is not solely a consequence of sarcopenia, as older adults may also show reduced voluntary activation in several muscle groups (Rozand et al., 2020). Furthermore, NMES combined with voluntary contraction has been reported to induce strength gains comparable to, or greater than, those obtained with voluntary training alone (Maffiuletti et al., 2013; Borzuola et al., 2023c). NMES can activate both motor and sensory pathways (Maffiuletti et al., 2013). Acute evidence indicates that, particularly when paired with voluntary contraction, NMES can facilitate corticospinal and spinal excitability and enhance somatosensory cortical responses, including during ankle dorsiflexion tasks (Borzuola et al., 2023c; Yamaguchi et al., 2023). This rationale is especially important in older adults, whose motor control is thought to become less automatic and more dependent on cognitive processes. Neuroimaging studies have shown broader and less lateralized prefrontal recruitment with aging, as described in the Hemispheric Asymmetry Reduction in Older Adults (HAROLD) model, as well as a posterior-to-anterior shift in activation, as described in Posterior-Anterior Shift in Aging (PASA). These patterns are often considered compensatory. In addition, older adults show increased prefrontal recruitment during motor planning (Cabeza, 2002; Davis et al., 2008; Berchicci et al., 2012). Consistent with these findings, cognitively demanding lower-limb tasks in older adults are associated with greater activation in prefrontal and motor planning regions. Aging is also linked to altered cortical control of ankle movement, while ankle-related tasks engage primary motor and somatosensory regions involved in force production and sensorimotor integration (Tung et al., 2023; Linortner et al., 2014; Yoshida et al., 2017; Borzuola et al., 2023a). Based on this rationale, we examined prefrontal regions related to cognitive motor control, including the DLPFC and frontal pole area (FPA), motor planning regions including the premotor/supplementary motor (PreM&SMC), primary motor cortex (M1; or abbreviated as PMC), and somatosensory processing regions (e.g., primary somatosensory cortex, SSC), during maximal ankle dorsiflexion in older adults.

A single NMES session may induce acute modulation of neural activity rather than lasting physiological adaptation. Such acute modulation may arise from afferent-driven sensorimotor integration and transient changes in sensorimotor excitability following stimulation, rather than consolidated cortical reorganization. Acute NMES has been shown to influence cortical dynamics and sensorimotor processing, and stimulation intensity/dose may influence the magnitude and direction of these short-term effects, supporting the plausibility of short-term modulation following peripheral stimulation (Borzuola et al., 2023b; Insausti-Delgado et al., 2021).

However, whether such acute modulation in healthy older adults is accompanied by measurable changes in ankle-related neuromuscular performance and task-evoked cortical activity remains unclear. This is a relevant question because lower-limb motor control in aging appears to rely more strongly on cognitive-motor and sensorimotor cortical resources, while most NMES studies in healthy older adults have focused on longer-term changes in strength, muscle mass, or muscle architecture rather than on immediate responses to a single session (Rahmati et al., 2021; Hansen et al., 2024; Jandova et al., 2020). This distinction is important because acute responses may reveal short-term neural and muscular modulation that is not apparent from long-term outcomes alone and may help explain how repeated NMES sessions lead to adaptation over time (Borzuola et al., 2023b; Blazevich et al., 2021).

Therefore, the specific problem addressed in the present study was the lack of integrated evidence on the immediate peripheral and cortical responses to a single NMES session in healthy older adults. To address this gap, we examined acute pre-to post-session changes after a single tibialis anterior NMES intervention using a multimodal design that combined isometric ankle dorsiflexion strength, muscle biomechanical properties, and whole-brain functional near-infrared spectroscopy during task performance. Based on previous acute NMES findings and aging-related changes in lower-limb cortical control, we hypothesized that a single NMES session would induce measurable short-term modulation of task-evoked cortical activity in prefrontal cognitive-motor control regions, motor-planning regions and somatosensory regions (SSC), accompanied by smaller but detectable changes in peripheral neuromuscular measures (Borzuola et al., 2023b; Blazevich et al., 2021; Tung et al., 2023; Linortner et al., 2014; Yoshida et al., 2017).

## 2 Materials and methods

### 2.1 Participants

Twenty-seven healthy older adult participants (66.2 ± 4.0 years old, 13 male/14 female) were enrolled in this study. All participants provided written informed consent in accordance with the Declaration of Helsinki. The study protocol was approved by the Ethics Committee of Beijing Rehabilitation Hospital (Approval Number: 2022bkky-061-001) and registered with the Chinese Clinical Trial Registry (Registration number: chictr2400093566). Healthy participants were recruited from nearby communities through poster advertisements. Full details can be found in Table 1. 

**TABLE 1:** General characteristics of participants.

Characteristic	Value
Sex (Male/Female)	13/14
Age (years)	66.2 ± 4.0
Height (cm)	163.1 ± 8.1
Weight (kg)	66.3 ± 11.6
BMI (kg/m^2^)	24.7 ± 2.5

Inclusion criteria: (a) Normal blood pressure and electrocardiogram (ECG); (b) Normal cognitive function (Montreal Cognitive Assessment [MoCA] score >26); (c) No history of severe musculoskeletal, cardiopulmonary, or nervous system diseases (e.g., stroke, Parkinson’s disease, myasthenia gravis); (d) No obvious visual or auditory impairments to ensure ability to accurately understand intervention instructions; (e) Voluntary participation and provision of written informed consent.

Exclusion criteria: (a) Abnormal skin conditions (e.g., damage, infection, rash, or scarring); (b) History of severe injuries to the spine or lower limbs (e.g., fractures, ligament rupture, or dislocation); (c) Participation in other clinical trials within the last month; (d) Other situations deemed inappropriate for participation by the researchers.

Sample size determination: The required sample size was estimated *a priori* based on a previously published NMES study in older adults with reduced muscle function (n = 16) reporting a mean percent improvement of 6.0% with a 95% confidence interval (±4.9) for peak torque after NMES (Kern et al., 2014). Because closely matched acute single-session NMES studies in healthy older adults were limited, the reference study was used as a pragmatic basis for estimating the effect size of the primary peripheral outcome. Based on the reported data, the estimated paired-samples effect size was Cohen’s d ≈ 0.65. *A priori* power analysis for a two-tailed paired t-test (α = 0.05, power = 0.80) indicated that 21 participants were required. To accommodate an anticipated 20% attrition rate and potential data-quality exclusions, we aimed to recruit 27 participants. Power analysis was performed using G*Power (version 3.1).

### 2.2 Experiment design

Participants entered a quiet, temperature-controlled room (22 °C–25 °C), received a standardized explanation of the procedures, and completed familiarization with the testing setup and required motor tasks. After fitting of the fNIRS cap and dynamometer stabilization, participants performed 5–10 submaximal ankle dorsiflexion trials (50%–75% maximum effort) to standardize task execution and reduce learning effects, followed by a 10-min seated rest period to stabilize physiological state before baseline assessment.

For the pre-intervention assessment, baseline physiological indicators (blood pressure, heart rate, and respiratory rate) were recorded, followed by assessment of muscle biomechanical properties of the right tibialis anterior, medial gastrocnemius, and lateral gastrocnemius using MyotonPro. Task-based fNIRS was then recorded during two sets of five maximal isometric ankle dorsiflexion contractions, with a 30-s resting baseline before the task and a 30-s recovery period after task completion. This task structure was selected to obtain repeated maximal efforts while maintaining tolerability in older adults and a block design compatible with fNIRS signal acquisition.

**FIGURE 1:**
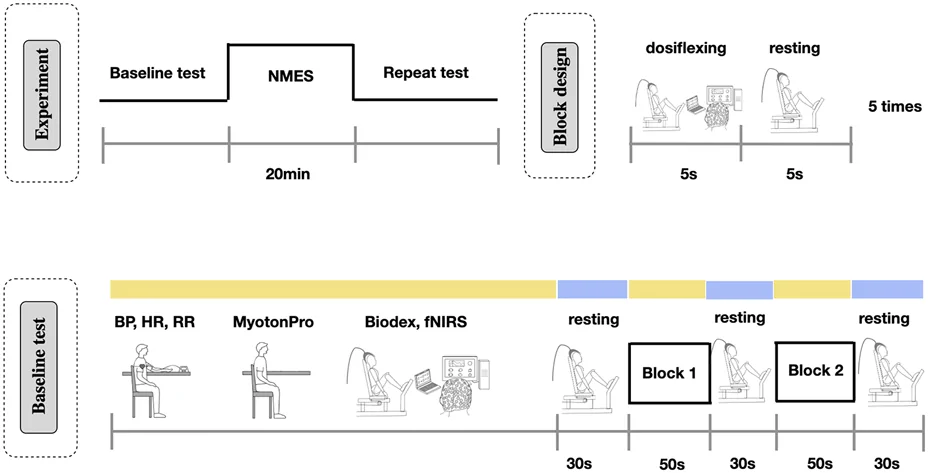
Experimental procedure.

Participants then underwent a 20-min NMES intervention applied to the right tibialis anterior (40 Hz, 2 s on/10 s off duty cycle, maximum tolerable intensity; see Section 2.3). For the post-intervention assessment, participants returned to the dynamometer, signal quality was re-verified, and after a standardized 10-min rest period the same physiological, MyotonPro, and task-based fNIRS assessments were repeated using the identical protocol. This post-intervention interval was used to standardize the measurement window after stimulation and to reduce the influence of immediate repositioning and transient physiological fluctuations. The overall experimental procedure is summarized in Figure 1.

Throughout the experimental session, researchers continuously monitored participants for signs of discomfort or adverse reactions. In case of abnormal conditions (such as palpitations, dizziness, local skin irritation, etc.), stimulation or testing was immediately stopped, and corresponding measures were taken.

### 2.3 Neuromuscular electrical stimulation

NMES was administered using a clinical-grade stimulator (Model S430, Nanjing Vishee Medical Technology Co., Ltd., Nanjing, China). Two self-adhesive electrodes (5 cm × 5 cm) were positioned over the right tibialis anterior muscle, with the cathode placed at the muscle belly (approximately one-third of the distance from the fibular head to the lateral malleolus) and the anode at the distal musculotendinous junction. The right tibialis anterior was selected as the standardized target muscle for all participants to standardize the tested limb, electrode placement, stimulation delivery, and dynamometer alignment across the sample.

The stimulation intensity was individualized for each participant. During a 2-min familiarization period, current was increased in 1 mA increments until a strong but tolerable muscle contraction was achieved, as confirmed by a verbal rating (“strong but tolerable”) and a VAS score ≤6/10 for discomfort. Stimulation parameters were set as follows: frequency 40 Hz, pulse width 400 μs, duration 20 min, and a duty cycle of 2 s on and 10 s off (on/off ratio: 1:5). These parameters were selected to balance evoked force production, participant tolerability, and feasibility in older adults. Prior work and parameter reviews suggest that frequencies in the 30–50 Hz range combined with pulse durations of approximately 400–600 μs are commonly used and appear effective for eliciting contractions while maintaining tolerability (Doucet and Griffin, 2013; Glaviano and Saliba, 2016; Nussbaum et al., 2017; Ramirez et al., 2023). The 20-min duration was selected as a practical single-session dose that could be implemented consistently within the experimental protocol.

### 2.4 Muscle strength assessment

Maximal ankle dorsiflexion strength was assessed using a Biodex System 4 dynamometer (System 4, Biodex Medical Systems, Shirley, NY, USA) under an isometric testing configuration. Participants were seated in an ergonomically adjusted chair, with the upper body secured by two crossed straps and the pelvis fixed with a single strap. The right ankle was positioned in the dynamometer footplate, ensuring the axis of rotation was aligned with the lateral malleolus and the ankle was set at 0° (neutral position) at the start of each contraction.

Participants then performed two sets of maximal isometric voluntary contractions (MIVCs), each set consisting of five contractions. Each contraction was held for 5 s, with a 5-s rest interval between contractions and a 30-s rest between sets. This protocol was chosen to obtain repeated maximal efforts while balancing signal robustness, participant tolerability, and task feasibility during simultaneous fNIRS recording in older adults. Prior to testing, participants were instructed to “push up as hard and fast as possible” and to maintain maximal effort throughout each contraction; standardized verbal encouragement was provided. Outcome measures included relative peak torque (Nm/kg; peak torque normalized to body mass), average peak torque (Nm; mean of peak torques across all contractions), and maximum peak torque (Nm; highest peak torque achieved).

### 2.5 Muscle bioechanical property assessment

Muscle biomechanical properties were evaluated using the MyotonPro device (Myoton AS, Tallinn, Estonia), a non-invasive handheld myotonometer. The MyotonPro has shown generally high reliability for assessing muscle mechanical properties. In the cited study, most reported ICC values ranged from 0.74 to 0.99 across muscles and parameters, although reliability was not uniformly high for every measure (Trybulski et al., 2024). Measurements were taken at three sites: the right tibialis anterior muscle belly (one-third of the distance from the fibular head to the lateral malleolus), the right medial gastrocnemius muscle belly, and the right lateral gastrocnemius muscle belly. At each site, ten consecutive measurements were performed with the coefficient of variation maintained below 3%, and the mean value was used for analysis. During assessment, participants were seated comfortably with the knee flexed at 90° and the ankle in a neutral position. The MyotonPro probe was placed perpendicular to the muscle surface with light, consistent pressure. The outcome measures included oscillation frequency (Hz; reflecting muscle tone), dynamic stiffness (N/m; reflecting resistance to deformation), and logarithmic decrement (reflecting muscle elasticity, with lower values indicating greater elasticity).

### 2.6 fNIRS system

#### 2.6.1 fNIRS data acquisition

**FIGURE 2:**
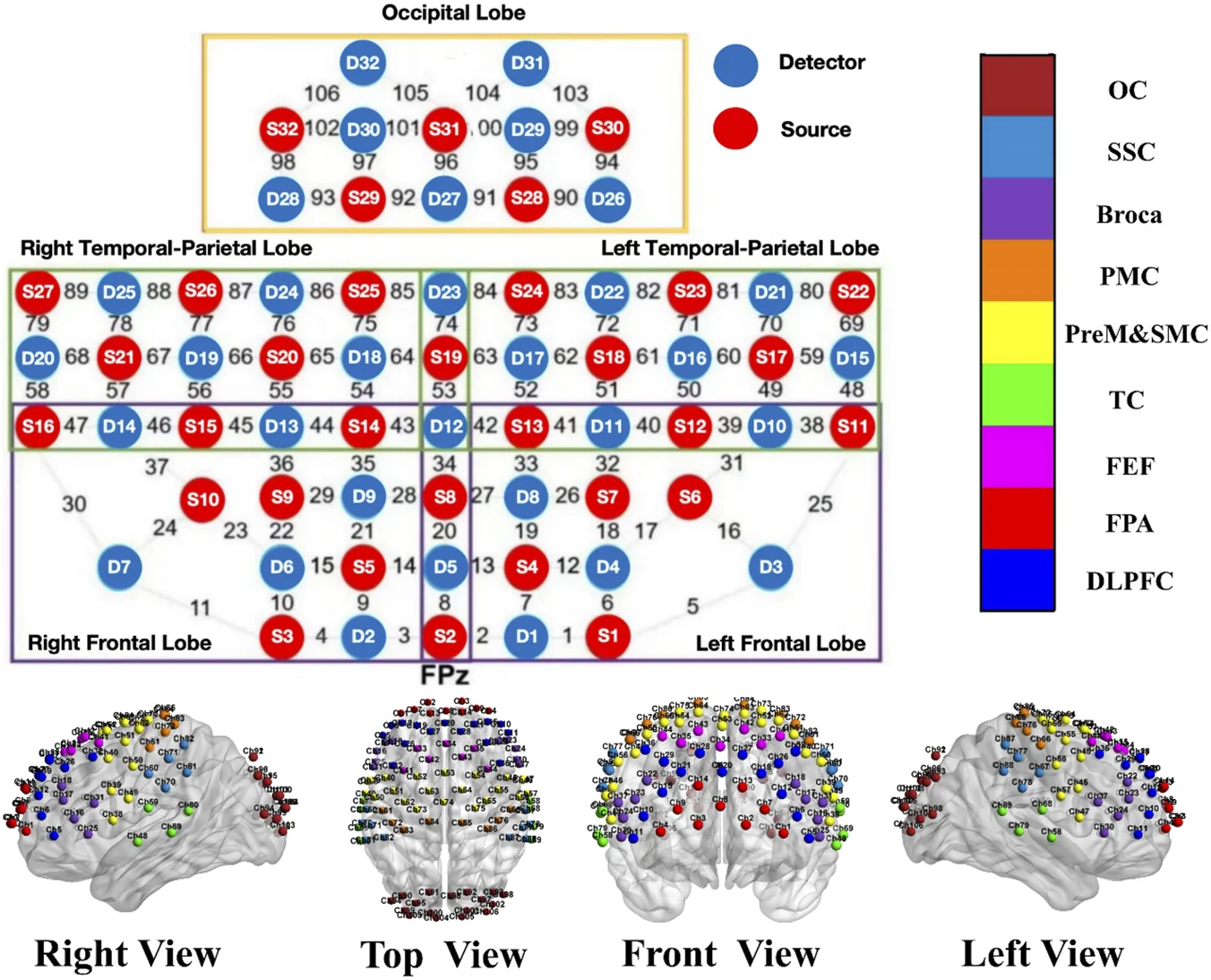
fNIRS Optode Montage and Channel Distribution. Red circles indicate light sources, blue circles indicate detectors, and the numbers between them indicate channel IDs. Channels were assigned to nine bilateral cortical ROIs using probabilistic anatomical registration. The complete channel-to-ROI mapping is provided in Supplementary Table S1. DLPFC, dorsolateral prefrontal cortex; FPA, frontal pole area; FEF, frontal eye field; TC, temporal cortex; PreM&SMC, premotor and supplementary motor areas; PMC, primary motor cortex; Broca, Broca’s area; SSC, primary somatosensory cortex; OC, occipital cortex.

Cortical activation was monitored using a 106-channel fNIRS device (BS-7000, Zilianhongkang, Wuhan, China) operating at a sampling rate of 20 Hz, with a fixed source–detector separation of 3 cm. The optode montage consisted of 64 optodes (32 light sources and 32 detectors) arranged on a soft cap according to the international 10–20 system (Kruppa et al., 2021), providing whole-cortex coverage (see Figure 2). Whole-cortex coverage was used because the ankle dorsiflexion task may engage not only sensorimotor regions but also broader cortical areas related to motor planning, attention, and sensory integration in older adults.

Channel-to-region-of-interest (ROI) mapping was performed using a validated probabilistic registration approach based on the manufacturer’s standardized optode template and Brodmann’s cytoarchitectonic atlas. Each channel was assigned to cortical ROIs based on the highest probability of anatomical correspondence. Nine bilateral cortical regions were defined: DLPFC, FPA, frontal eye field (FEF), temporal cortex (TC), PreM&SMC, primary motor cortex (PMC), Broca’s area (Broca), SSC, and occipital cortex (OC). For statistical analyses, each region was further subdivided into left and right hemispheres, resulting in 18 ROIs (e.g., L-DLPFC, R-DLPFC). The complete probabilistic channel-to-ROI mapping is provided in Supplementary Table S1. While this method does not account for individual anatomical variability, it provides a reasonable approximation for group-level analyses in healthy adults and has been validated in previous fNIRS studies (Tsuzuki and Dan, 2014).

Neuronal activity was quantified by converting optical density changes into relative concentration changes of oxygenated hemoglobin (HbO), deoxygenated hemoglobin (HbR), and total hemoglobin (HbT) using the modified Beer–Lambert law (Kocsis et al., 2006). HbO was selected as the primary outcome measure given its higher signal-to-noise ratio, greater sensitivity to task-evoked cerebral blood flow changes, and positive association with the blood-oxygen-level-dependent (BOLD) signal reported in fMRI studies (Chen et al., 2022). After confirming stable signal quality across all channels, the formal testing protocol commenced.

#### 2.6.2 fNIRS data processing and analysis

fNIRS data were processed in MATLAB R2020b (The MathWorks, Inc., Natick, MA, USA) using NIRS_KIT (Version 3.0) and SPM toolboxes. Raw light intensity data were first converted to hemoglobin concentration changes (HbO and HbR) using the modified Beer–Lambert law. The preprocessing pipeline was designed to minimize physiological noise and motion artifacts while preserving task-related signals. Linear detrending was applied to reduce slow baseline drift, followed by motion artifact correction using the time-derivative distribution repair (TDDR) method (Fishburn et al., 2019), which identifies and interpolates motion-contaminated segments based on temporal derivative thresholds. An infinite impulse response (IIR) band-pass filter with a passband of 0.01–0.1 Hz was then applied to attenuate very low-frequency drift and higher-frequency physiological noise components such as respiration (∼0.2–0.3 Hz) and cardiac pulsation (∼1 Hz).

At the individual level, a general linear model (GLM) was applied to estimate task-evoked hemodynamic responses for each channel. The preprocessed HbO time series was modeled as a linear combination of task regressors convolved with a canonical hemodynamic response function (HRF), yielding participant-specific beta coefficients that quantify the magnitude of task-evoked activation for each channel in each session (pre- and post-intervention). ROI-level beta values were subsequently computed by averaging the channel-level beta coefficients within each anatomically defined ROI, providing a summary measure of regional activation.

### 2.7 Statistical analysis

Statistical analyses were performed using IBM SPSS Statistics 25.0 (IBM Corp., Armonk, NY, USA) and MATLAB R2020b. Normality of continuous variables was evaluated using the Kolmogorov–Smirnov test. Data conforming to a normal distribution are presented as mean ± standard deviation (SD) and were analyzed using paired-sample t-tests. Data not conforming to a normal distribution are presented as median (interquartile range) and were analyzed using the Wilcoxon signed-rank test. Unless otherwise specified, statistical significance was set at p < 0.05, and all tests were two-tailed.

#### 2.7.1 Peripheral outcomes (muscle strength and muscle mechanical properties)

Pre-versus post-intervention differences were evaluated for relative peak torque, average peak torque, maximum peak torque, oscillation frequency, dynamic stiffness, and logarithmic decrement using paired-sample t-tests or Wilcoxon signed-rank tests as appropriate. Effect sizes were reported as Cohen’s d to quantify the magnitude of pre–post changes, with d = 0.2, 0.5, and 0.8 representing small, medium, and large effects, respectively (Cohen, 1988).

#### 2.7.2 ffNIRS outcomes (group-level inference)

Group-level inference was conducted using a repeated-measures mixed-effects modeling framework to account for within-participant dependence. The analyzed variables were task-evoked HbO beta estimates at both the channel level (106 channels) and ROI level (18 ROIs). Two contrasts were evaluated: (1) within-session activation (pre or post) was assessed by testing whether beta estimates differed from zero (against-zero inference), and (2) intervention-related modulation was assessed by comparing post-intervention versus pre-intervention beta estimates. To reduce potential systemic physiological influences, heart rate, mean arterial pressure (MAP), and respiration rate were included as covariates in the group-level models. Mean arterial pressure (MAP) was calculated as MAP = (SBP +2 × DBP)/3. Effect sizes (Cohen’s d) and 95% confidence intervals were reported.

#### 2.7.3 Brain-behavior correlation analysis

To examine the relationship between cortical activation changes and muscle strength improvement, Spearman’s rank correlation analyses were performed between changes in task-evoked HbO beta values (Δβ = post-intervention β–pre-intervention β) and changes in maximum peak torque (ΔTorque = post-intervention torque–pre-intervention torque) at the ROI level. Partial correlations controlling for baseline heart rate, MAP, and respiration rate were also computed to account for individual differences in physiological state. Given the exploratory nature of these analyses, both uncorrected and FDR-corrected results are reported.

#### 2.7.4 Correction for multiple comparisons

To control for Type I error inflation due to multiple testing, false discovery rate (FDR) correction (Benjamini and Hochberg, 1995) was applied separately for channel-wise analyses (106 channels), ROI-wise analyses (18 ROIs), and brain–behavior correlation analyses (18 ROIs). FDR-corrected significance was defined as q < 0.05. Results that did not survive FDR correction but showed uncorrected p < 0.05 were reported as exploratory findings and interpreted with appropriate caution.

## 3 Results

### 3.1 Physiological parameters

Physiological parameters were monitored before and after the NMES intervention and remained within normal ranges throughout the study (Table 2). Systolic and diastolic blood pressure decreased significantly after NMES (p = 0.015, d = 0.499; p = 0.037, d = 0.422, respectively), whereas respiratory rate, heart rate, and heart rate variability showed no significant changes (all p > 0.05). All 27 participants completed the full protocol without premature termination. The mean stimulation intensity applied during NMES was 28.5 ± 6.2 mA, with a range of 18–42 mA across participants.

**TABLE 2:** Physiological parameters before and after intervention.

Parameter	Pre-NMES	Post-NMES	t	p	Cohen’s d
Systolic BP (mmHg)	133.63 ± 16.85	126.74 ± 14.50	2.593	0.015*	0.499
Diastolic BP (mmHg)	85.52 ± 8.98	82.07 ± 8.73	2.192	0.037*	0.422
Respiratory rate (breaths/min)	17.00 ± 1.66	16.70 ± 1.51	1.093	0.285	0.210
Heart rate (bpm)	80.70 ± 11.29	77.59 ± 11.04	1.513	0.142	0.291
HRV (ms)	26.89 ± 7.53	29.30 ± 8.73	−1.484	0.150	0.286

BP, blood pressure; HRV, heart rate variability. All values are mean ± SD. *p < 0.05.

### 3.2 Muscle strength and physical properties test indicators

Following NMES, isometric strength of the tibialis anterior increased significantly across all three measures (Table 3). Average peak torque showed the largest improvement (p < 0.001, d = 0.861), with significant increases also observed in relative peak torque (p = 0.045, d = 0.383) and maximum peak torque (p = 0.014, d = 0.476).

**TABLE 3:** Muscle strength and bioechanical properties before and after NMES.

Parameter	Pre-NMES	Post-NMES	t	p	Cohen’s d
Isometric strength					
Relative peak torque (N·m/kg)	53.85 ± 14.48	58.02 ± 15.06	−2.096	0.045*	0.383
Average peak torque (N·m)	29.85 ± 7.85	34.81 ± 8.61	−4.716	<0.001**	0.861
Maximum peak torque (N·m)	35.12 ± 8.87	38.58 ± 10.52	−2.609	0.014*	0.476
TA mechanical properties					
Oscillation frequency (Hz)	18.28 ± 2.55	18.48 ± 3.10	−0.346	0.732	0.063
Dynamic stiffness (N/m)	381.10 ± 66.17	380.73 ± 78.51	0.024	0.981	0.004
Logarithmic decrement	1.01 ± 0.13	0.98 ± 0.18	0.959	0.346	0.175

TA, tibialis anterior. All values are mean ± SD., All paired t-tests: df = 26 (n = 27).

*p < 0.05, **p < 0.001.

In contrast, no significant changes were observed in the passive mechanical properties of the tibialis anterior, including oscillation frequency, dynamic stiffness, and logarithmic decrement (all p > 0.05). Similarly, mechanical properties of the medial and lateral gastrocnemius muscles showed no significant pre-post differences (all p > 0.05; Supplementary Table S2), indicating that the acute effects of NMES were specific to active force production rather than passive tissue properties.

### 3.3 Cortical activation status

#### 3.3.1 Pre-intervention task-evoked cortical activation

**FIGURE 3:**
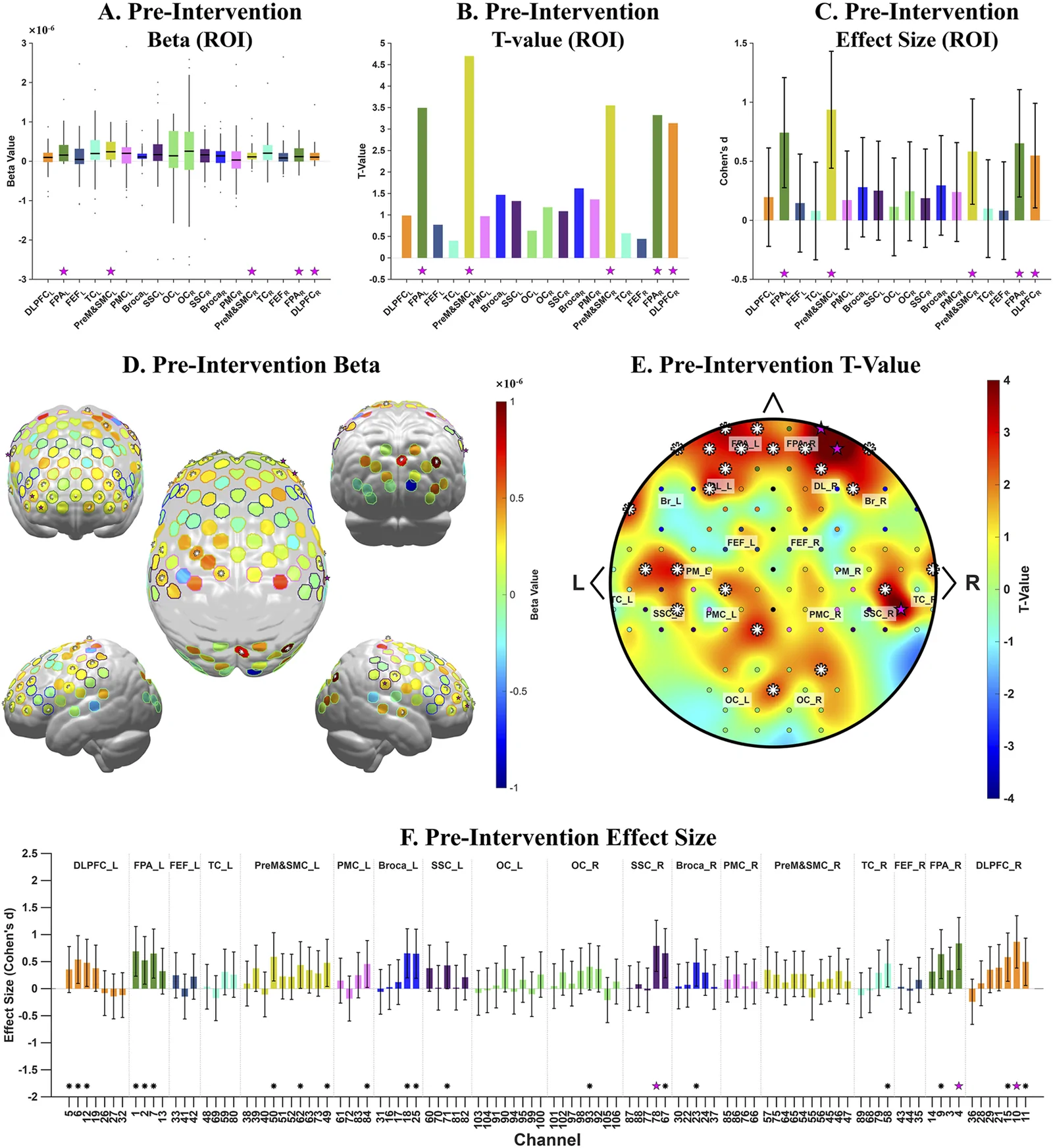
Pre-intervention Task-evoked Cortical Activation During Ankle Dorsiflexion (Pre). **(A–C)** ROI-level summaries of task-evoked activation (tested against zero). **(A)** Boxplots display the median HbO beta estimate for each ROI (central line = median; box = interquartile range; whiskers = distribution range). **(B)** ROI-level t-values for the against-zero contrast. **(C)** ROI-level effect sizes (Cohen’s d) shown as mean ±95% confidence interval (CI). **(D–F)** Channel-level visualizations of task-evoked activation. **(D)** 3D cortical maps of channel-wise HbO beta estimates (warm colors indicate greater task-evoked activation). **(E)** 2D topographic map of channel-wise t-values for the against-zero contrast. **(F)** Channel-wise effect sizes (Cohen’s d) shown as mean ±95% CI. Significance markers: ^★^ indicates FDR-corrected significance (q < 0.05) and * indicates uncorrected significance (p < 0.05, exploratory).

During the pre-intervention ankle dorsiflexion task, significant task-evoked cortical activation was observed across a distributed network (Figure 3). At the ROI level, FDR-corrected effects (q < 0.05) were identified in the left and right PreM&SMC, bilateral FPA, and right DLPFC. The channel-level pattern was broadly consistent with the ROI-level findings.

#### 3.3.2 Post-intervention task-evoked cortical activation

**FIGURE 4:**
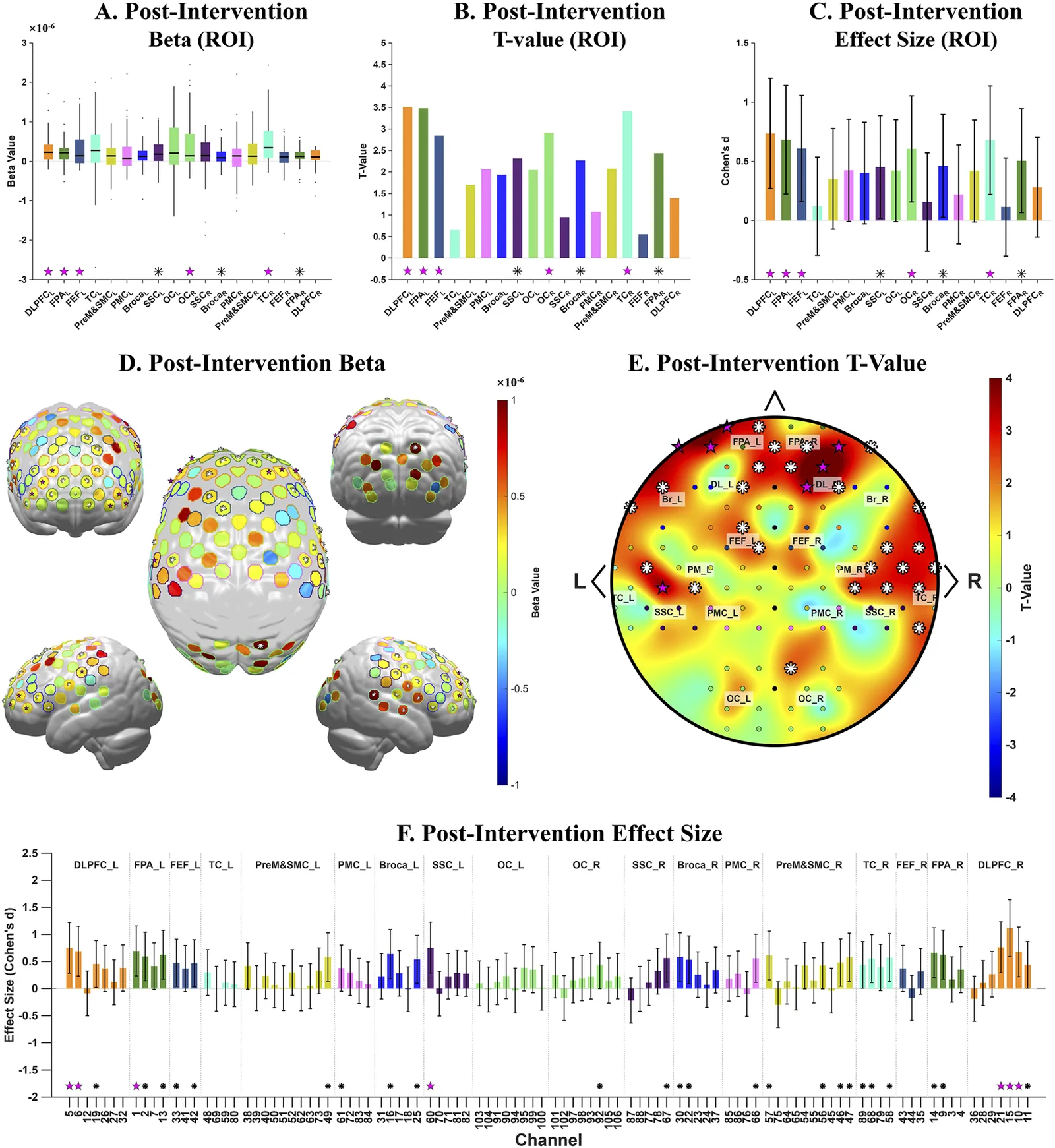
Post-intervention Task-evoked Cortical Activation During Ankle Dorsiflexion (Post). **(A–C)** ROI-level summaries of task-evoked activation (tested against zero). **(A)** Boxplots display the median HbO beta estimate for each ROI (central line = median; box = interquartile range; whiskers = distribution range). **(B)** ROI-level t-values for the against-zero contrast. **(C)** ROI-level effect sizes (Cohen’s d) shown as mean ±95% confidence interval (CI). **(D–F)** Channel-level visualizations of task-evoked activation. **(D)** 3D cortical maps of channel-wise HbO beta estimates (warm colors indicate greater task-evoked activation). **(E)** 2D topographic map of channel-wise t-values for the against-zero contrast. **(F)** Channel-wise effect sizes (Cohen’s d) shown as mean ±95% CI. Significance markers: &starf; indicates FDR-corrected significance (q < 0.05) and * indicates uncorrected significance (p < 0.05, exploratory).

Following NMES, task-evoked cortical activation remained evident during the same task and involved a wider set of activated regions (Figure 4). At the ROI level, FDR-corrected activation (q < 0.05) was observed in the left DLPFC, left FPA, right TC, right OC, and left FEF. The channel-level findings provided partial support for the ROI-level results.

#### 3.3.3 Differences in cortical activation: Pre-vs. post-intervention

**FIGURE 5:**
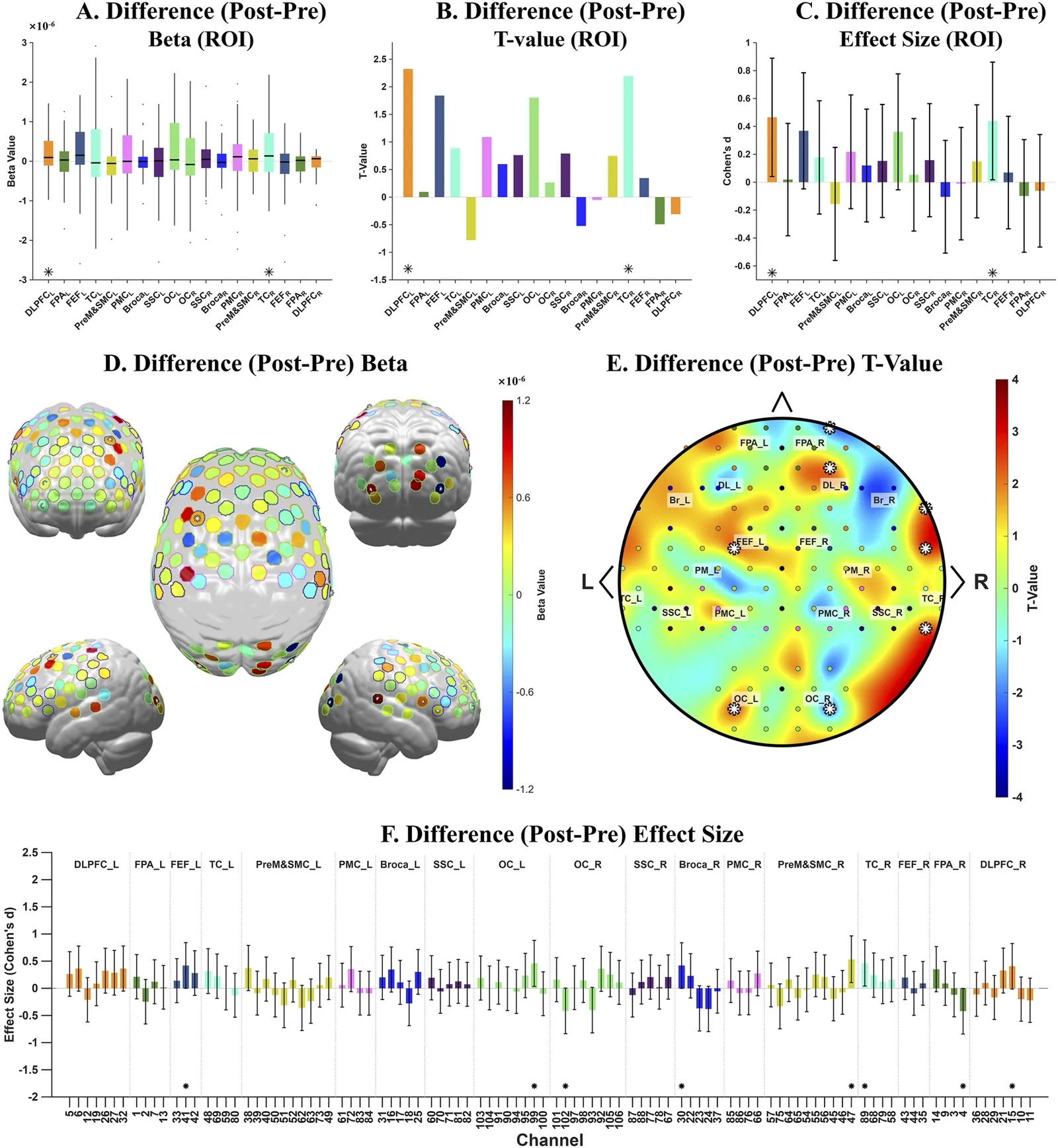
Pre–post Differences in Task-evoked Cortical Activation (Post–Pre). **(A–C)** ROI-level summaries of post–pre changes in task-evoked activation. **(A)** Boxplots display the median post–pre HbO beta difference for each ROI (central line = median; box = interquartile range; whiskers = distribution range). **(B)** ROI-level t-values for the post–pre contrast. **(C)** ROI-level effect sizes (Cohen’s d) shown as mean ± 95% confidence interval (CI). **(D–F)** Channel-level visualizations of the post–pre contrast. **(D)** 3D cortical maps of channel-wise post–pre HbO beta differences (warm colors indicate Post > Pre; cool colors indicate Post < Pre). **(E)** 2D topographic map of channel-wise t-values for the post–pre contrast. **(F)** Channel-wise effect sizes (Cohen’s d) for the post–pre contrast shown as mean ± 95% CI. Significance markers: ^★^ indicates FDR-corrected significance (q < 0.05) and * indicates uncorrected significance (p < 0.05, exploratory).

A mixed-effects model was used to evaluate post-pre differences in task-evoked cortical activation (Figure 5). No ROI- or channel-level effects survived FDR correction (q < 0.05); therefore, the following findings are reported as exploratory (uncorrected p < 0.05) and should be interpreted cautiously. A localized increase in activation (Post > Pre) was observed in the right PreM&SMC (Ch_47: p = 0.0105, d = 0.534). At the ROI level, upward trends were found in the left DLPFC (p = 0.0247, d = 0.465) and right TC (p = 0.0334, d = 0.439). Additional localized channel-level increases were observed in the right TC, left OC, left FEF, right Broca’s area, and right DLPFC, whereas decreases were observed in the right FPA and right OC.

### 3.4 Brain-behavior correlations

**FIGURE 6:**
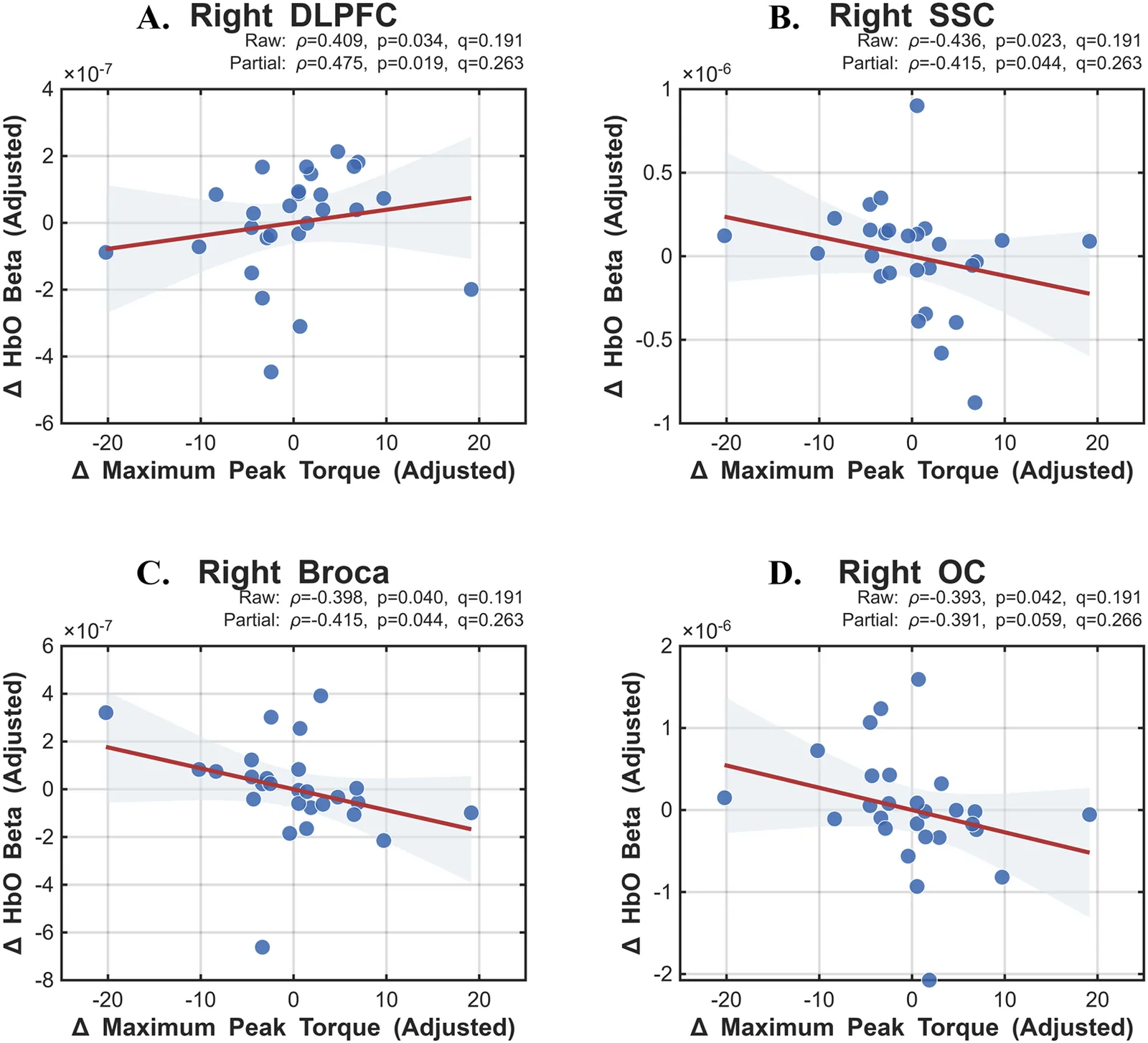
Brain-behavior Correlations Between Cortical Activation Changes and Muscle Strength Improvement. Scatter plots show partial correlations (controlling for baseline heart rate, mean arterial pressure, and respiration rate) between change in Maximum Peak Torque (X-axis) and change in task-evoked HbO beta values (Y-axis) in **(A)** Right DLPFC **(B)** Right SSC **(C)** Right Broca’s area, and **(D)** Right OC. Each blue dot represents one participant. Both axes display adjusted residuals after regressing out baseline physiological covariates. Red line: linear fit; shaded area: 95% CI. Statistics (top right): Raw = Spearman correlation without covariates; Partial = Spearman partial correlation controlling for baseline covariates (ρ: correlation coefficient; p: uncorrected p-value; q: FDR-corrected q-value).

Correlation analyses between changes in cortical activation and muscle strength improvement revealed several exploratory associations, although none survived FDR correction (Figure 6). After controlling for baseline physiological parameters (heart rate, mean arterial pressure, and respiration rate), partial Spearman correlations showed a positive association in the right DLPFC (ρ = 0.475, p = 0.019, q = 0.263) and negative associations in the right SSC (ρ = −0.415, p = 0.044, q = 0.263), right Broca’s area (ρ = −0.415, p = 0.044, q = 0.263), and right OC (ρ = −0.391, p = 0.059, q = 0.266). Raw correlations without covariate adjustment showed similar patterns. All other ROIs showed no significant correlations (all p > 0.05).

## 4 Disscussion

### 4.1 Overview of main findings

The main finding of this study was that a single session of NMES was associated with an immediate improvement in ankle dorsiflexion strength in healthy older adults, as reflected by increases in relative peak torque, average peak torque, and maximum peak torque. By contrast, no measurable changes were found in the passive mechanical properties of the tibialis anterior over the same period. These results suggest that a single NMES session can acutely facilitate voluntary force production without detectable short-term changes in passive tissue mechanics.

At the cortical level, the ankle dorsiflexion task elicited activation in cognitive-motor and sensorimotor regions in both the pre-intervention and post-intervention sessions. No pre–post cortical differences remained significant after FDR correction. However, exploratory analyses suggested localized changes involving prefrontal, premotor/supplementary motor, somatosensory, and temporo-occipital regions. These findings support a cautious interpretation. NMES may acutely improve neuromuscular performance, whereas the cortical results should be interpreted cautiously and do not provide firm evidence of intervention-specific cortical change.

### 4.2 Peripheral strength facilitation

The immediate increase in dorsiflexion torque after NMES is physiologically plausible and is consistent with previously described motor unit recruitment mechanisms associated with electrical stimulation (Carson and Buick, 2021; Maffiuletti, 2010). In the present study, NMES was delivered at 40 Hz and at the highest intensity tolerated by each participant. Compared with lower stimulation frequencies such as 20 Hz, this setting may be more favorable for force production. Previous work has suggested that 40 Hz can effectively induce relatively strong evoked contractions (Doucet and Griffin, 2013). Although such evidence should not be directly extended across different populations, it offers useful context for understanding the acute strength response observed here.

One possible explanation for the increase in torque is that NMES recruits motor units differently from voluntary activation. During voluntary contraction, recruitment generally follows the Henneman size principle, with smaller motor units activated before larger ones (Henneman et al., 1965). Electrical stimulation, in contrast, can more directly activate peripheral motor axons and produce a more synchronous recruitment pattern (Gregory and Bickel, 2005). This pattern may increase force output over a short period of time, and synchronous discharge across a larger motor unit pool may also allow more muscle fibers to reach peak contraction at the same time, which may contribute to the transient increase in peak torque observed after NMES (Del Vecchio et al., 2018). These mechanisms offer a reasonable physiological explanation for acute strength enhancement without the need to assume immediate structural changes in passive tissue properties.

### 4.3 Task-evoked cortical activation

Interpretation of the cortical findings requires consideration of the task itself. In older adults, repeated maximal isometric ankle dorsiflexion likely depends not only on motor output, but also on sustained attention, regulation of effort, movement planning, and continuous sensorimotor monitoring. From this perspective, the involvement of prefrontal, premotor/supplementary motor, and somatosensory regions in both sessions is not surprising. It is in line with previous work showing that motor planning and cognitively demanding lower-limb tasks in older adults place substantial demands on cognitive-motor control processes (Berchicci et al., 2012; Cabeza, 2002; Davis et al., 2008; Tung et al., 2023).

Within this task-related framework, the involvement of DLPFC, FPA, and PreM&SMA was especially notable, which is compatible with the demands of sustained cognitive control and motor planning during repeated maximal contractions. Concurrent activation of SSC further suggests an important contribution of proprioceptive feedback to force regulation. Overall, this distributed pattern points to the complexity of cortical control during maximal ankle dorsiflexion in older adults. Because the task required repeated maximal voluntary contraction of the right ankle, the widespread bilateral activation likely reflects, at least in part, the neural demands of task performance itself.

### 4.4 Pre–post differences in task-evoked cortical activation

With respect to intervention-related effects, the key observation is that no pre–post cortical differences survived FDR correction. This suggests that cortical changes specifically attributable to the NMES session were limited relative to the statistical threshold applied and should not be overstated. For this reason, the post-intervention cortical findings are best regarded as exploratory.

Within that exploratory context, post-intervention trends were most apparent in regions linked to cognitive-motor control and sensorimotor processing, particularly DLPFC, FPA, PreM&SMA, SSC, and temporo-occipital areas. These findings raise the possibility that the post-intervention task was performed under a somewhat altered neural context compared with the pre-intervention session. One possible explanation is that acute NMES influenced the neural conditions under which the post-intervention task was performed, perhaps through altered afferent input and related changes in task-related engagement. Another possibility is that repeated performance of the maximal dorsiflexion task was accompanied by changes in attentional allocation, task strategy, familiarization, or other session-related factors. The present design does not allow these alternatives to be separated.

This issue is especially important in a single-session study. Because the post-test was completed shortly after stimulation and no sham condition was included, it is difficult to distinguish effects that were specific to the intervention from those related to task repetition, state changes, or familiarization. The present findings therefore should not be interpreted as evidence of established cortical plasticity. Rather, they suggest that acute NMES may be associated with subtle changes in task-evoked cortical recruitment, which should be tested further in sham-controlled designs.

Fatigue is unlikely to be a sufficient explanation for the cortical trends observed here. Although fatigue cannot be completely excluded, it was not directly quantified in the present study, and torque increased rather than decreased after the intervention. In this context, altered task state, sensory feedback, attentional demands, and familiarization appear more plausible contributors to the post-intervention cortical pattern than fatigue alone.

### 4.5 Brain–behavior associations

The exploratory brain–behavior analyses suggested that participants with larger torque gains tended to show greater increases in right DLPFC activation, whereas negative associations were observed in SSC, Broca’s area, and occipital cortex. One possible interpretation is that acute performance improvement may be accompanied by different patterns of task-related cortical engagement during repeated maximal efforts. At this stage, however, these associations should be viewed only as exploratory observations.

At the same time, none of the brain–behavior correlations survived FDR correction. These findings should therefore be treated strictly as exploratory observations rather than mechanistic evidence. At this stage, it is reasonable only to suggest that individuals may achieve acute strength gains through partly different neural strategies, and this possibility will need to be tested in larger sham-controlled studies. Future work should also consider whether baseline fitness, prior exposure to NMES, sensory responsiveness, or sex contributes to between-participant variability in responsiveness to NMES.

### 4.6 Limitations

Several limitations should be acknowledged. First, this was a single-session within-participant pre–post study without a sham control and with a modest sample size. This limits causal interpretation and makes it difficult to distinguish intervention-specific effects from non-specific influences such as task repetition, attentional shifts, motivation, or familiarization. In line with this limitation, no pre–post cortical effects survived FDR correction, and all cortical change patterns should therefore be interpreted as exploratory.

Second, systemic physiological fluctuations may have affected the HbO signals. Although heart rate, mean arterial pressure, and respiration rate were included as covariates, residual systemic effects cannot be fully excluded. In addition, short-separation channels were not available to regress superficial hemodynamic signals (Goodwin et al., 2014; Zhou et al., 2020). Motion artifacts were addressed using TDDR, but preprocessing cannot completely remove all non-neural components (Fishburn et al., 2019). ROI assignment was based on a standard template rather than individualized optode digitization, which may have introduced spatial uncertainty.

Third, only two task blocks were used, which may have reduced sensitivity for detecting subtle cortical effects. Finally, sex-related differences were not formally examined. Because sex may influence neuromuscular function and cortical responses, the absence of sex-specific or sex-adjusted analyses should be recognized as a limitation (Mehta and Rhee, 2021). The present study was not designed or statistically powered to support formal subgroup analyses by sex. Future studies with larger samples should determine whether sex moderates the acute response to NMES.

## 5 Conclusion

In healthy older adults, a single session of NMES applied to the tibialis anterior increased ankle dorsiflexion strength immediately after stimulation, while passive muscle mechanical properties did not show a measurable short-term change. Cortical activation during the task was observed both before and after the intervention, which likely reflects the demands of repeated maximal ankle dorsiflexion itself. Although some localized post-pre differences were seen in the exploratory analyses, they did not survive correction for multiple comparisons. Therefore, the present findings support an acute effect of NMES on neuromuscular performance, whereas the cortical results should be interpreted cautiously and remain to be clarified in sham-controlled and longitudinal studies.

## Data Availability

The raw data supporting the conclusions of this article will be made available by the authors, without undue reservation.
