# Correction: Acute changes in ankle dorsiflexor strength and fNIRS-derived cortical activation following a single session of neuromuscular electrical stimulation in healthy older adults

**DOI:** 10.3389/fragi.2026.1975276

**Published:** 2026-09-03

**Authors:** Yingqi Li, Luyi Wang, Congxiao Wang, Hujun Wang, Shaoting Zhang, Anda Xiu, Shengxuan Duan, Yingpeng Wang, Shuyan Qie

**Affiliations:** 1 Department of Rehabilitation, Beijing Rehabilitation Hospital, Capital Medical University, Beijing, China; 2 School of Beijing Rehabilitation, Capital Medical University, Beijing, China

**Keywords:** ankle dorsiflexion, cortical activation, functional near-infrared spectroscopy, muscle strength, neuromuscular electrical stimulation, older adults

The revised complete funding statement is as follows: This work was supported by Beijing High‐Level Innovation and Entrepreneurship Talent Support Program (“Qingmiao”) Project, Grant No. G202522175, and the National Key R&D Program of China (Ministry of Science and Technology of China), Special Project on Active Health and Technological Responses to Population Aging, Grant No. 2022YFC3600501, 2022YFC3600500.

The original version of this article has been updated.

