# Supplementary material for: Acute changes in ankle dorsiflexor strength and fNIRS-Derived cortical activation following a single session of neuromuscular electrical stimulation in healthy older adults
Source: Front Aging. 2026 Jun 15;7:1726632. doi: 10.3389/fragi.2026.1726632 (PMC13310975; doi:10.3389/fragi.2026.1726632)
Supplement: Supplementary file 1 [file Table2.docx]

**Supplementary Table S2. Gastrocnemius Mechanical Properties Before and After NMES**

| **Parameter** | **Pre-NMES** | **Post-NMES** | **t** | **p** | **Cohen's d** |
| --- | --- | --- | --- | --- | --- |
| **Medial Gastrocnemius** | | |  |  |  |
| Oscillation Frequency (Hz) | 11.52 ± 1.46 | 11.82 ± 1.37 | −1.096 | 0.282 | 0.200 |
| Dynamic Stiffness (N/m) | 206.03 ± 39.20 | 211.83 ± 37.41 | −0.771 | 0.447 | 0.141 |
| Logarithmic Decrement | 1.62 ± 0.27 | 1.56 ± 0.34 | 0.914 | 0.368 | −0.167 |
| **Lateral Gastrocnemius** | | |  |  |  |
| Oscillation Frequency (Hz) | 13.17 ± 1.82 | 13.28 ± 2.34 | −0.329 | 0.744 | 0.060 |
| Dynamic Stiffness (N/m) | 256.33 ± 40.60 | 254.13 ± 59.97 | 0.221 | 0.826 | −0.040 |
| Logarithmic Decrement | 1.59 ± 0.24 | 1.66 ± 0.31 | −1.109 | 0.277 | 0.202 |

*All values are mean ± SD. All paired t-tests: df = 26 (n = 27).*
